# Supplementary material for: Joint coding of shape and blur in area V4
Source: Nat Commun. 2018 Jan 31;9:466. doi: 10.1038/s41467-017-02438-8 (PMC5792439; doi:10.1038/s41467-017-02438-8)
Supplement: Supplementary file 1 — Supplementary Information [file 41467_2017_2438_MOESM1_ESM.pdf]

Supplementary Information

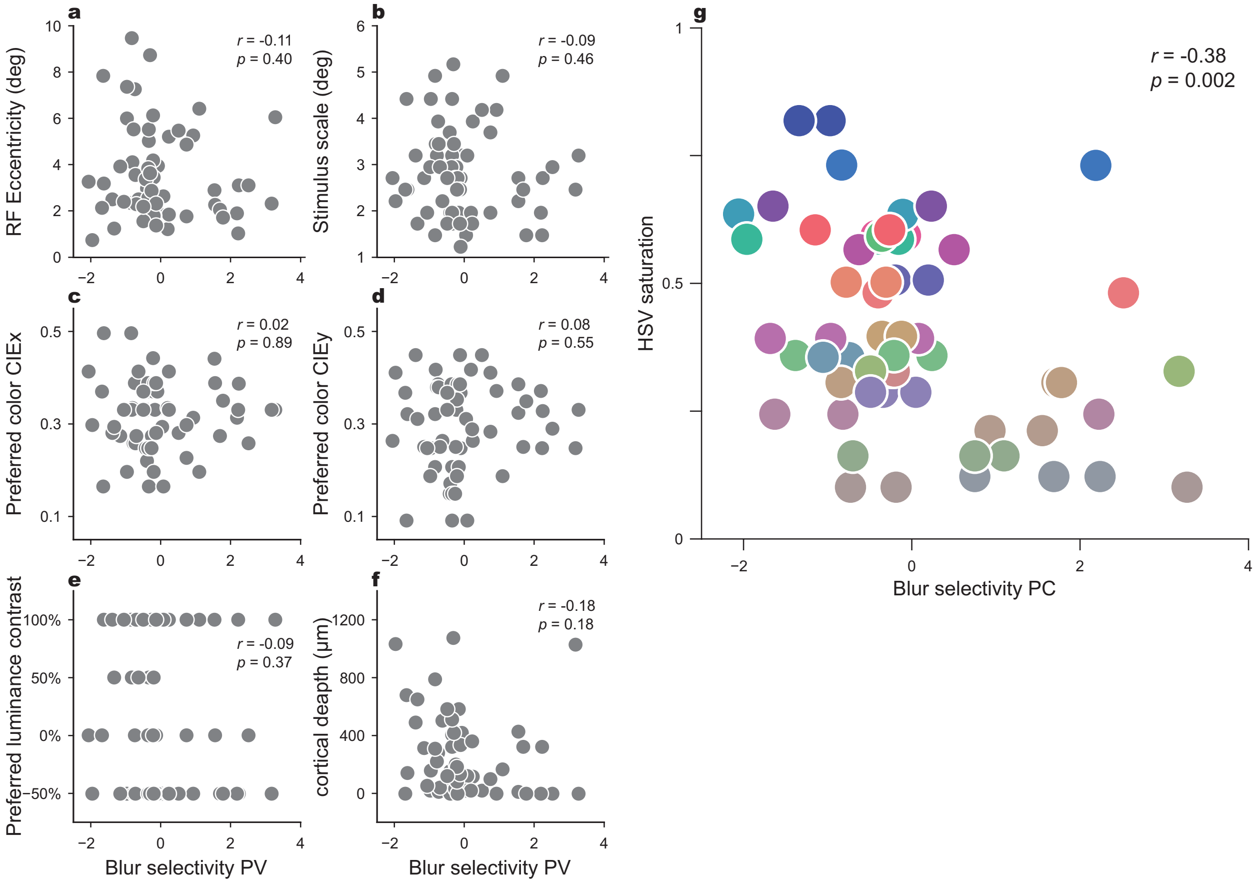

Supplementary Figure 1: Physiological properties of blur selectivity across neurons. (a-g) Receptive field and neuronal data observed from each neuron in our dataset, plotted as a function of the principle value of blur selectivity (see Fig. 2e). (a) Eccentricity, (b) preferred size, (c-d) chromaticity, (e) luminance contrast, or (f) approximate cortical depth are not significantly correlated with blur selectivity. (g) A significant correlation ($p = 0.002$) is found between blur selectivity and saturation of preferred colour in the HSV space, indicating neurons selective for intermediate blur tend to prefer shape stimuli defined by a low chromatic contrast. Each neuron is colored according to preferred hue as given in (c,d).
